# Role of microRNAs in Lung Carcinogenesis Induced by Asbestos

**DOI:** 10.3390/jpm11020097

**Published:** 2021-02-03

**Authors:** Rakhmetkazhy Bersimbaev, Olga Bulgakova, Akmaral Aripova, Assiya Kussainova, Oralbek Ilderbayev

**Affiliations:** Department of General Biology and Genomics, Institute of Cell Biology and Biotechnology, L.N. Gumilyov Eurasian National University, Nur-Sultan 010008, Kazakhstan; aripova001@gmail.com (A.A.); assya.kussainova@gmail.com (A.K.); oiz5@yandex.ru (O.I.)

**Keywords:** carcinogenesis, lung cancer, microRNA, asbestos exposure

## Abstract

MicroRNAs are a class of small noncoding endogenous RNAs 19–25 nucleotides long, which play an important role in the post-transcriptional regulation of gene expression by targeting mRNA targets with subsequent repression of translation. MicroRNAs are involved in the pathogenesis of numerous diseases, including cancer. Lung cancer is the leading cause of cancer death in the world. Lung cancer is usually associated with tobacco smoking. However, about 25% of lung cancer cases occur in people who have never smoked. According to the International Agency for Research on Cancer, asbestos has been classified as one of the cancerogenic factors for lung cancer. The mechanism of malignant transformation under the influence of asbestos is associated with the genotoxic effect of reactive oxygen species, which initiate the processes of DNA damage in the cell. However, epigenetic mechanisms such as changes in the microRNA expression profile may also be implicated in the pathogenesis of asbestos-induced lung cancer. Numerous studies have shown that microRNAs can serve as a biomarker of the effects of various adverse environmental factors on the human body. This review examines the role of microRNAs, the expression profile of which changes upon exposure to asbestos, in key processes of carcinogenesis, such as proliferation, cell survival, metastasis, neo-angiogenesis, and immune response avoidance.

## 1. Introduction

Lung cancer is one of the most frequent forms of cancer and is one of the leading causes of death from malignant neoplasms worldwide [1,2]. Approximately 1.8 million new cases of lung cancer are diagnosed annually in the world, and more than 1.5 million people die from this disease every year. According to experts, the number of deaths from lung cancer will increase to 3 million in 2035 [3]. The five-year survival rate for lung cancer ranges from 5% to 17% (average 15%), depending on the stage of the disease at the time of its diagnosis. The American Cancer Society estimated that there were about 234,000 new cases and about 154,000 deaths for lung cancer in the United States in 2018 [4].

Lung cancer is commonly associated with smoking and exposure to the carcinogenic components of tobacco smoke. About 90% of lung cancers in men and 80% in women are caused by tobacco smoking. Smoking causes about 25% of deaths among women and men [5]. Research estimated that for men and women between the ages of 25 and 70, the mortality rate among smokers was about three times higher than among those who had never smoked [6]. The increased mortality of smokers is mainly associated with neoplasms and respiratory diseases [7]. The life expectancy of smokers is more than 10 years shorter than those who have never smoked [2].

Carcinogenic environmental factors, such as air pollution and air emissions from fuel combustion as well as environmental exposure to radon, asbestos, certain metals (such as chromium, cadmium, and arsenic), and some organic chemicals, also contribute to the development of lung cancer [8]. Environmental carcinogens can cause specific genetic and epigenetic changes in lung tissue, leading to aberrant functions of lung cancer oncogenes and tumor suppressor genes [9]. Research also found that about 25% of lung cancer cases occur in people who have never smoked [7]. Thus, we can say that lung cancer today is an important medical problem, and progress in the treatment of this group of cancers can be achieved by improving our understanding of the molecular basis and biology of the tumor, especially at the level of the cells that trigger the tumor process [9].

## 2. Asbestos as a Carcinogenic Factor of the Environment

One of the important environmental carcinogen associated with lung cancer is asbestos [10,11]. Asbestos fibers are naturally occurring silicate mineral fibers that have long been used in industry due to their exceptional properties, such as tensile strength, low thermal conductivity, and relative resistance to chemical attack. For these reasons, asbestos has been used for insulation in buildings and as an ingredient in a variety of products, such as roofing shingles, water pipes, and fire-retardant coatings, as well as clutch and brake pads, density rings, and vehicle supports. Asbestos is used as an additive for asphalt concrete to increase the stability of the road surface.

There are six types of asbestos mineral fibers: chrysotile (white asbestos), crocidolite (blue asbestos), amosite (brown or gray asbestos), anthophyllite, tremolite, and actinolite [10]. The main types of asbestos are chrysotile (white asbestos), the spiral-shaped, most common form of asbestos, and crocidolite (blue asbestos). Amphiboles (crocidolite, amosite, anthophyllite, tremolite, and actinolite) are straight rod-shaped fibers that have a needle-like appearance, while serpentines (for example, chrysotile) are curved fibers.

Numerous industrial workers are exposed to asbestos dust, as is a significant proportion of the urban population associated with the extraction, processing, and industrial use of asbestos. Exposure to asbestos fibers is strongly associated with the development of malignant mesothelioma and lung cancer [12,13]. Asbestos-related lung disease is a major health problem worldwide [13,14].

All identified forms of asbestos have been classified as human carcinogens by the International Agency for Research on Cancer. According to World Health Organization (WHO) estimates, 125 million people worldwide are exposed to asbestos, and the use of this substance can cause the development of not only lung cancer but also ovarian and laryngeal cancer as well as mesothelioma [15,16]. Exposure to asbestos also causes diseases such as asbestosis (pulmonary fibrosis) and pleural plaques thickening and effusion. It is estimated that around 110,000 people die annually from lung cancer, mesothelioma, and asbestosis as a result of exposure to asbestos.

Between 5% and 7% of all lung cancer cases in the world have high levels of asbestos as the cause of the disease, mainly due to occupational exposure [17]. Roughly half of the deaths from occupational cancer in workers in the asbestos industry are due to asbestos [18].

There are many industries where workers deal with asbestos. Possible ways of contact with this material are as follows: “Primary”, extraction, sorting, grinding; “Industrial”, the production of asbestos itself and products from it; “Construction”, all kinds of construction and installation work, for example, the installation of boiler equipment, laying pipelines; and “Ecological”, the industrial emissions of asbestos industries, which are dangerous for people living in the neighborhood; the destruction of buildings constructed with the use of asbestos and asbestos-containing materials, without observing the relevant standards; and uncontrolled removal, the release of asbestos waste and dust into the natural environment [10,19].

In addition, it is estimated that several thousand deaths annually may be caused by the use of asbestos and asbestos-containing materials in the home; for example, asbestos was widely used at one time in the manufacture of ironing boards. It has also been shown that simultaneous exposure to tobacco smoke and asbestos fibers significantly increases the risk of lung cancer—the more a person smokes, the higher the risk [20,21].

Respirable fibers are the main source of exposure to asbestos [17,21]. With prolonged inhalation of asbestos dust, pneumoconiosis (silicatoses) and chronic dust bronchitis develop, the clinical picture of which has features due to the physicochemical properties of the corresponding types of dust. In industrial environments, exposure to mixed dust containing silicates and free silica is possible. Due to the fibrous structure of asbestos, dust, in addition to its fibrosing effect, causes more pronounced mechanical damage to the mucous membrane of the respiratory tract and lung tissue than other types of industrial dust [17].

The accumulation of asbestos fibers in the lungs leads to fibrosis, inflammation, and carcinogenesis, although the specific effects depend on the dose and type of fiber inhaled [20].

The mechanism of destruction that occurs in the lungs as a result of exposure to asbestos is determined by the efficiency of removing fibers from the cells of the respiratory tract. Longer fibers can penetrate deeper into the respiratory tract and are cleared more slowly than short ones and are associated with a higher carcinogenic potential [11,13,21]. Other elements, such as iron (which can account for up to 30% of the weight of asbestos fibers), embedded in the surface of the fibers can also enhance the pathogenic effects associated with asbestos.

### 2.1. Asbestos and Lung Diseases

Asbestos causes asbestosis and malignant neoplasms through molecular mechanisms that are not fully understood. The side effects of asbestos generally fall into three categories: pleural disease, lung parenchymal disease, and neoplastic disease. Effects on the pleura include pleural effusions, plaques, and diffuse pleural thickening. In the parenchyma, rounded atelectasis, fibrous cords, and asbestosis are observed. Exposure to asbestos can lead to neoplastic diseases, such as lung cancer pleural mesothelioma, peritoneal mesothelioma, and bronchogenic carcinoma [20]. The mechanisms of action underlying asbestosis, lung cancer, and mesothelioma appear to differ depending on the fiber type, lung clearance, and genetics. The picture of asbestos-induced carcinogenesis is complex with many types and results of molecular aberrations that can arise from exposure [22].

When asbestos dust interacts with human cells, asbestos silicates attract and bind to cations; in the lungs, asbestos fibers hold ions on their surface, thereby contributing to the leaching of the cellular environment [23]. These processes can generate reactive oxygen species (ROS), which initiate the processes of cell and DNA damage and explain the genotoxic effect of asbestos [24,25]. There are at least three sources of ROS production when exposed to asbestos, including (a) fiber surface reactivity, (b) release from immune cells, especially alveolar macrophages, and (c) mitochondrial ROS released from immune and other target cells, such as lung epithelial cells and mesothelial cells [26].

The high iron content in some asbestos fibers and the tendency of asbestos to adsorb iron in vivo have led to the assumption that iron-induced Fenton reactions also contribute to an increase in ROS, inflammation, and carcinogenesis [20]. Likely, it is ROS and the oxidative stress developing as a result of their action that underlie the damage to the lung tissue [13]. Oxidative stress can promote apoptosis, gene mutations, chromosomal aberrations, and, ultimately, cell transformation [17,20]. Inflammation, as mentioned above, is another important source of ROS production, given that all forms of asbestos activate the generation of ROS by neutrophils and alveolar macrophages of rodents and humans during the so-called frustrated phagocytosis, a process that is accompanied by the release of cytokines, chemokines, proteases, and growth factors contributing to the development of an inflammatory response [14,21].

In addition, research demonstrated that oxidative stress caused by exposure to asbestos dust can activate signaling pathways, including mitogen-activated protein kinases, nuclear factor kB (NF-kB), and activator protein 1, which control cell proliferation, apoptosis, and the inflammatory response [14]. The accumulated data strongly suggest that ROS generated from the mitochondria of key target cells mediate pulmonary asbestos toxicity. Thus, Carter and colleagues demonstrated an important role in the production of H_2_O_2_ by the mitochondria of alveolar macrophages using an animal model of asbestosis [27,28].

A high level of apoptosis, in turn, can trigger the development of inflammatory processes in lung tissue due to another type of free-circulating nucleic acids—free-circulating mitochondrial DNA (fc mtDNA). The fc mtDNA copy number changes in different types of malignant neoplasias, including lung cancer [29]. Fc mtDNA, through TLR-9 receptors, can also mediate the activation of the NF-kB signaling pathway and, as a consequence, develop aseptic inflammation [28,29]. However, the role of fc mtDNA in the pathogenesis of lung cancer induced by exposure to asbestos dust remains unexplored, despite the fact that, as mentioned above, the mitochondria themselves are directly involved in the development of the cellular and molecular effects of asbestos.

### 2.2. The Role of microRNAs in Lung Cancer Carcinogenesis

**Figure 1:**
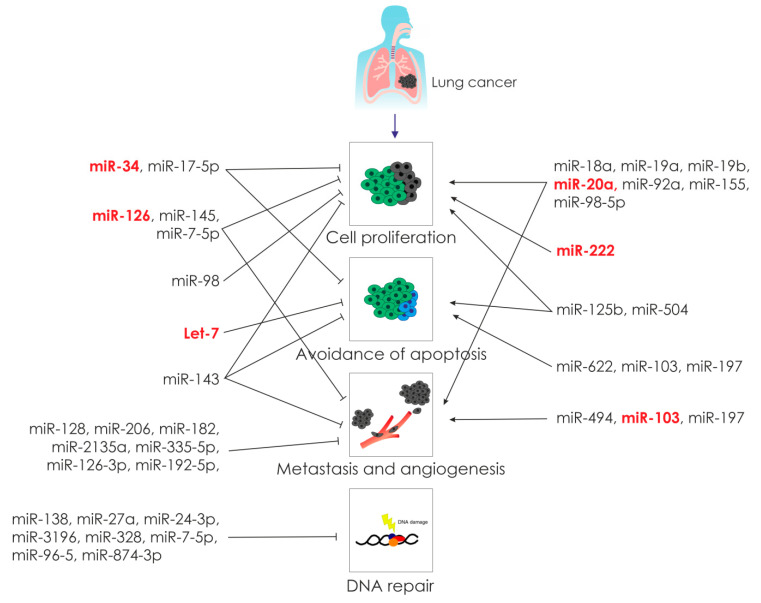
Cellular aspects of lung carcinogenesis with microRNA regulations (microRNAs whose expression profile changed under the influence of asbestos, are highlighted in red).

Another type of free-circulating nucleic acids involved in the process of carcinogenesis is microRNA [30,31]. MicroRNAs are small noncoding RNAs that are involved in the regulation of target genes at the posttranscriptional level. MicroRNAs can covalently bind to complementary sequences in the 3′ UTR region of the mRNA and thereby inhibit translation. It is known that microRNAs control many cellular processes such as proliferation, differentiation, and cell death (Figure 1).

According to various estimates, microRNAs can control the expression of up to 50% of human genes [32]. MicroRNAs are found in tissue cells, but they are also found in extracellular areas, in blood plasma, and in other body fluids. MicroRNAs have also been found in platelets, erythrocytes, and nucleated blood cells. In the extracellular fluids of the body, they are carried in small membrane vesicles (exosomes), forming complexes with high density lipoproteins or carrier proteins.

At present, microRNAs are isolated into a separate class, most widely represented today, of short noncoding RNAs. According to the latest revision (January 2019), the mirbase.org database contains information on 38,589 hairpin precursors and 48,860 mature microRNAs from 271 species [32].

To date, a large body of evidence has been accumulated on the involvement of microRNAs in the carcinogenesis of various malignant neoplasias: malignant pleura, mesothelioma, prostate cancer, hematological malignant neoplasms, glioblastoma, and others. Subsequently, many studies have shown that the expression of certain microRNAs closely correlates with the development and progression of lung cancer. Research found that microRNAs are directly involved in many types of cancer, including lung cancer. MicroRNAs can not only be regulators of oncogenes but can themselves be regulated by oncogenes or suppressors of oncogenes [33,34].

Although microRNA is not tissue-specific, tumor cells develop a unique genetic profile during oncogenesis. The profiles of circulating microRNAs are different for each microenvironment and stage of cancer progression. Analysis of these microRNA profiles provides a better understanding of tumor pathogenesis and cancer origins. As tumors alter the normal concentration of circulating microRNAs, research suggested that these nucleotides can be used for early diagnosis, staging, follow-up, and the assessment of therapeutic responses and therapy outcomes in certain types of human cancer, including lung cancer [30,33]. MicroRNAs playing the role of oncogenes (OncomiR) and oncosuppressors in the development of lung cancer are shown in Table 1 and Table 2.

#### 2.2.1. MicroRNA and Cell Proliferation in Lung Cancer

MicroRNAs play an important role in the control of cell proliferation [35] (Figure 1). An example is the human mir-17 cluster, consisting of six microRNAs: hsa-miRs-17-5p, -18, -19a, -19b, -20, and -92. This cluster is located at 13q31, which is often amplified in several types of lymphomas and solid tumors. Research demonstrated that the oncogenic protein c-MYC binds directly to the genomic locus encoding these microRNAs to activate their transcription. In addition, two microRNAs of this cluster, hsa-miR-17-5p and hsa-miR-20, target the transcription factor E2F1, which regulates the expression of proapoptotic proteins in the cell, thereby avoiding apoptosis and increasing cell proliferation [36].

An example of microRNA oncosuppressors is the let-7 family, whose members target *ras* oncogenes (*H-ras*, *K-ras*, and *N-ras*) [37]. As *ras* overexpression is a key oncogenic event in lung cancer, the involvement of let-7 in the pathogenesis of this disease is beyond doubt. Indeed, let-7 expression in lung cancer cells is significantly reduced as compared to normal tissue. In addition, the RAS protein levels in bronchial epithelial cells are inversely proportional to the let-7 levels, which is consistent with microRNA-mediated translational repression of the *ras* gene [37]. The expression of let-7 in the lung carcinoma cell line A549 directly suppresses the growth of cancer cells in vitro [37], illustrating the effectiveness of targeted antitumor therapy using this microRNA.

Another oncosuppressive microRNA is miR-126. Studies have shown that miR-126 can inhibit the proliferation of NSCLC through the suppression of EGFL7 and PTEN/PI3K/AKT signaling pathways [38,39]. In addition, decreased expression of miR-126 was associated with adhesion, migration, and invasion of NSCLC cells due to an increase in the Crk protein [40]. Hence, miR-126 may function as an important regulatory gene in the development of NSCLC. Research found that miR-145 is involved in the regulation of tumor cell proliferation by disabling the signaling pathways RAS/ERK, PI3K/AKT, ERK5/c-MYC, and p68/p72/β-catenin [41,42].

Recent meta-analysis demonstrated that miR-155 may be a potential biomarker for lung cancer detection. Experiments on an animal model showed that mice that were artificially injected with miR-155 exhibited proliferation of lung tumors [43]. In addition, it was found that overexpression of miR-155-5p significantly extended the malignant phenotype of lung cancer cells, including cell growth, colony formation, migration, invasion, and antiapoptotic effects [44,45].

A recent study indicated that miR-222 overexpression was related to NSCLC risk [46]. It was shown that miR-222 promotes the growth of non-small cell cancer cell lines by targeting oncosuppressor p27, which controls the cell cycle progression at G1 [47].

#### 2.2.2. MicroRNA and Apoptosis in Lung Cancer

MicroRNAs can also have antiproliferative and proapoptotic activities (Figure 1). These molecules function in the cell as tumor suppressors. The main regulator of apoptosis in the cell is the p53 protein. Recent studies indicated the relationship between the profile of certain microRNAs and the expression level of the *TP53* gene. It was shown that the change in the microRNA profile after p53 induction occurs in the direction of an increase in the content of microRNA-34a, 34b, and 34c [48]. The level of these microRNAs increased in response to genotoxic stress with the involvement of p53 both in vitro and in vivo.

The transcription of microRNA-34a, -34b, and -34c at both loci is directly activated by p53. Studies have shown that members of the hsa-miR-34 family inhibit the expression of several targets involved in cell cycle regulation, such as cyclin E2 and cyclin-dependent kinases 4 and 6 (CDK4 and CDK6), and BCL2 [48]. Interestingly, some *TP53* mutations, which were previously associated with oncogenic progression, suppress the expression of some microRNAs [48]. p53 can also serve as a target for some types of microRNAs. Research showed that miR-504 can target the mRNA of p53. Ectopic expression of miR-504 decreases the p53 protein level, which inhibits p53-dependent apoptosis and arrest of the cell cycle in the G1 phase [49]. hsa-miR-125b targets p53 and proapoptotic proteins Bak1 and Puma, which leads to the inhibition of apoptosis [50].

Studies demonstrated the radioprotective role of some types of microRNA. In vitro studies using the WI-38 human fibroblast line showed that the mature form of hsa-miR-155 inhibited radiation-induced premature “cellular senescence” [51]. In this regard, scientists assumed that some microRNAs can determine the resistance of tumor cells to radiation therapy and be used as a predictive biomarker to monitor the effectiveness of cancer treatment.

The suppression of apoptosis may underlie this effect. Thus, the overexpression of hsa-miR-622 in colon cancer cells inhibits the Rb protein, thus inactivating the Rb-E2F1-P/CAF complex, whose participation is a key moment in the activation of proapoptotic genes [52].

In the development and homeostasis process, apoptosis plays a significant role. There are two ways of separating the apoptotic process: external and internal. A series of cascading proteases are triggered by each pathway, and cell death occurs eventually. Tumor cells have the capacity to inhibit apoptosis and thus support the survival of cells. In response to a broad range of endogenous and exogenous signals, including DNA damage, hypoxia, ribosome biogenesis inhibition, food starvation, ribonucleotide triphosphate depletion, and oncogene activation (C-Mus, RAS, and E2F-1), p53 is activated. p53 causes cell cycle arrest, apoptosis, or aging, depending on the cell type, environmental background, and degree of stress, to avoid the spread of damaged cells that may potentially become cancerous [53].

In tumorigenesis, microRNAs are important apoptosis regulators, and cancer cells can control microRNAs in oncogenesis to regulate cell survival. miR-103 expression in the carcinoma tissues of NSCLC patients was found to be increased. After knockdown of miR-103, the number of apoptotic cells increased, the expression level of the proapoptotic protein Bax increased, and the level of the antiapoptotic protein Bcl-2 decreased in A549 and H23 cell lines. In addition, the level of FBW7 protein was increased after the miR-103 knockdown. F-box and WD repeat domain-containing 7 (FBW7) is a tumor inhibiting protein that can inhibit the emergence and development of numerous types of tumors by regulating the cycle, differentiation, apoptosis, proliferation, invasion, and migration of tumor cells [54].

The induction of apoptosis in wild-type p53 cells is the responsibility of two BH3-only NOXA and BMF proteins as new miR-197 targets, identifying miR-197 as a main survival factor in NSCLC. The inhibition of miR-197 is, therefore, proposed as a new therapeutic approach toward lung cancer [55]. The miR-197-3p profile was elevated in the tissues of patients with lung adenocarcinoma. The inhibition of miR-197-3p expression led to increased apoptosis through the activation of caspase-3/7 in lung adenocarcinoma cells. The overexpression of miR-197-3p stimulated proliferation and did not block apoptosis of human bronchial epithelial cells, thus suggesting that miR-197-3p expression has an important role in malignant neoplasms [56].

The content of miR-17 was decreased in T2–T4 pathologic stage NSCLC tissues and SK-MAS-1, A549, SPCA-1, H460, H1229, and HCC827 cell lines. Li et al. demonstrated that miR-17-5p inhibited proliferation and caused apoptosis of H460 NSCLC cells, inhibiting TGFßR2, which was significantly increased in NSCLC tissues and cell lines [57]. An experiment was also conducted in paclitaxel-resistant lung cancer cells. Paclitaxel exerts a cytotoxic effect, inducing apoptosis [58]. However, in drug-resistant cancer, tumor cells overcome this cytotoxic effect of paclitaxel and become resistant to apoptosis. Increased miR-17-5p expression and paclitaxel treatment induced apoptotic cell death in lung cancer cells [59].

An increased expression profile of miR-486 protected against PM2.5-induced cell apoptosis in cell lines A549 [60]. Studies have shown that PM2.5 treatment can cause cell apoptosis, cell necrosis, autophagy, DNA damage, mitochondrial damage, and gene mutations in respiratory tract tissues [61]. Recently, the effect of propofol in the H1299 and H1792 lung cancer cell lines and the role of miR-486 have been studied.

When treated with propofol in cell lines, the expression level of miR-486 increased, and cell viability, when combined with propofol with an inhibitor of miR-486, was increased compared to the control and propofol group. The authors indicated that increased expression of miR-486 may contribute to the antitumor activity of propofol [62]. Increased expression of the miR-486-5p profile blocked cell proliferation and invasion by repressing GAB2 in non-small cell lung cancer [63].

The level of expression of miR-98 was reduced simultaneously in both tissues and cell lines A549 and H12999 [63]. Tumor size, TNM level, lymph node metastasis, and survival in pancreatic adenocarcinoma were associated with decreased miR-98 expression [64]. High miR-98 expression patients showed longer average survival than low miR-98 expression patients [65]. Inhibited miR-98 activated PAK1 (P21-activated protein kinase 1), a biomarker of pulmonary cancer, which promotes NSCLC cell proliferation, migration, and invasion [66].

#### 2.2.3. MicroRNA and Angiogenesis in Lung Cancer

Different pro- or antiangiogenic factors manipulate angiogenesis, a key step in tumor growth and metastasis. By modulating the expression of essential angiogenic factors, microRNAs have recently been shown to modulate the angiogenic processes (Figure 1). The function of microRNAs derived from tumors in controlling tumor vascularization remains to be explained [67].

The targeting of PTEN and subsequent activation of the Akt/eNOS pathway mediates the angiogenic effect of miR-494. Importantly, coculture experiments showed that, through a microvesicle-mediated pathway, a lung cancer cell line, A549, secreted and delivered miR-494 into endothelial cells. In addition, in response to hypoxia, the expression of miR-494 was induced in tumor cells, possibly through the HIF-1 alpha-mediated mechanism [68]. 

A mimic miR-128 significantly suppressed the expression of vascular endothelial growth factor (VEGF)-C. The overexpression of miR-128 in NSCLC cells and human endothelial vein umbilical cells caused a reduction expression of VEGF-A, vascular endothelial growth factor receptor 2, and VEGFR-3, essential factors critical for cancer angiogenesis and lymphangiogenesis, and slightly declined the phosphorylation of extracellular signal-regulated kinase, phosphatidylinositol 3-kinase, and p38 signaling pathways [69]. miR-206 decreased the angiogenic efficiency of NSCLC by inhibiting the 14-3-3ζ/STAT3/HIF-1α/VEGF pathway [70]. miR-135a decreased angiogenesis-related factors VEGF, bFGF, and IL-8 in A549 cells by inhibiting IGF-1 [71]. 

#### 2.2.4. MicroRNA and Metastasis in Lung Cancer

A large percentage of cancer deaths are associated with the metastasis of primary tumors [72]. The invasion of cancer cells and the formation of metastases is a clinically significant process. The molecular genetics and epigenetic mechanisms of this process are the least studied at the moment.

The spread of tumor metastasis is a complex multistage process that includes the invasion of cancer cells into the lumen of a blood or lymphatic vessel, migration, localization in a new place, the growth of metastases, and the formation of additional blood vessels to feed the metastatic foci.

Lung cancer is often diagnosed at an advanced stage with the formation of metastases [73]. Tumors of the lung have preferred organs for metastasis—the brain, bones, liver, and adrenal glands. Other organs may be affected in the terminal stage of the disease. It has been observed that various histological types of lung cancer typically metastasize to specific organs. In SCLC, metastasis is found mainly in the liver and brain, and adenocarcinoma in the brain and bones, [74,75], while NSCLC affects mainly the bones, brain, adrenal glands, liver, and lymphatic vessels [76].

Molecular genetics and epigenetic changes during metastasis are associated with the epithelial–mesenchymal transition, which is activated by the activity of internal factors—KRAS, Her2, MET, and EGFR, [77,78,79,80,81] as well as external factors TGF-β, EGF, HGF, PDGF, TNF-α, and IL-6 [81]. The role of microRNAs in metastasis was first reported by Ma et al., who found that the overexpression of miR-10b promotes the formation of lung metastases in breast cancer [82]. miR-10b also exhibits oncogenic activity and is involved in the metastasis of NSCLC (Figure 1). The expression of miR-10b in lymphatic vessel metastases was significantly higher compared to the primary tumor in the lungs [83].

The role of miR-646 in the development of metastases in renal cancer [84] and gastric cancer [85] is ambiguous. MiR-646 exhibits tumor suppressive properties in pancreatic cancer [86]. TRIM44 activates the AKT/mTOR signal pathway to induce melanoma progression by stabilizing TLR4. Zhang et al. showed that miR-646 can inhibit proliferation and invasion and can suppress the EMT of NSCLC cells in mice. The FGF2 and CCND2 genes are targets for miR-646. Overexpression of miR-646 significantly suppressed the expression of mRNA and protein FGF2 and CCND2 in NSCLC cell lines [87].

Li et al. showed that overexpression of miR-182 promoted the expression of E-cadherin, which led to the inhibition of EMT. MiR-182 also suppresses AKT phosphorylation and accumulation of the Snail transcription factor, which initiates EMT in lung cancer cells. As mentioned above, the hepatocyte growth factor HGF activates EMT by activating the Met signaling pathway, which, as a result, increases the invasive and metastatic potential of cells. It has been shown that miR-182 can directly bind to Met, thereby negatively regulating Met expression and reducing lung tumor metastasis [88].

miR-7-5p inhibits the proliferation, migration, and invasion of tumor cells by regulating the expression of genes associated with EMT both in vitro and in vivo. miR-7-5p also suppresses NSCLC metastasis by acting on NOVA2 [89], which presumably disrupts the angiogenesis of the resulting metastases and inhibits their further growth [90].

Liao et al. found a decrease in the miR-206 levels in NSCLC. The oncosuppressive mechanism of miR-206 is that this microRNA inhibits the development of NSCLC metastases through the negative regulation of the actin-binding protein coronin 1C (CORO1C) [91]. CORO1C knockdown significantly reduced the ability of cells to grow and metastasize in gastric [92] and breast cancer [93].

Decreased expression of miR-335-5p and increased expression of the ROCK1 oncogene was observed in NSCLC metastases to lymphatic vessels. The overexpression of miR-335-5p led to inhibition of TGF-β1-mediated EMT in NSCLC as a result of downregulation of ROCK1 [94]. ROCK1 is also known to reduce PTEN activation/phosphorylation and then phosphorylate PI3K/AKT, which leads to the phosphorylation of FAK tyrosine kinase, which plays a key role in cell adhesion and in stimulating cell migration and invasion [95].

#### 2.2.5. MicroRNAs Participating in Epithelial–Mesenchymal Transition (EMT)

A significant decrease in the miR-126-3p expression in NSCLC cell lines and tissues and an increase in the miR-126-3p levels suppressed the migration and invasion of cancer cells (Figure 1). Studies demonstrated that miR-126-3p inhibited NSCLC cell growth and metastasis by acting on chemokine receptor 1 (CCR1) [96]. miR-192-5p was significantly reduced in patients with metastatic lung cancer [97]. This microRNA reduces the migration and invasion of lung cancer by inhibiting TRIM44, which induces EMT by activating the AKT/mTOR signaling pathway [98]. This mechanism of metastasis was also observed in melanoma [99] and esophageal cancer [100].

miR-625 is a suppressor in NSCLC. There was a decrease in miR-625 expression found in NSCLC tissues, which likely contributes to tumor progression and metastasis due to EMT activation via the PI3K/AKT/Snail signaling pathway [101]. Overexpression of miR-652-3p was found in tumor tissues of patients with NSCLC and was significantly higher in patients with lymph node metastases. The probable mechanism of metastasis is the binding of miR-652-3p mRNA of the Lgl1 protein, which promotes cell adhesion and inhibits cell migration by suppressing the expression of MMP2 and MMP14 and re-expression of E-cadherin [102].

Xia et al. noted that miR-143 suppressed NSCLC cell proliferation, induced apoptosis, and suppressed migration and invasion in vitro. Limk1 has been identified as a direct target for miR-143 [103]. Decreases in the Limk1 levels were also noted in prostate [104] and breast [105] cancers. miR-98-5p inhibits translation of the messenger RNA encoding TGFBR1, which is involved in the regulation of cellular processes, including motility, differentiation, adhesion, division, and apoptosis. Decreased TGFBR1 levels led to proliferation, migration, and invasion in A549 and H1299 cell lines [106].

#### 2.2.6. MicroRNAs and DNA Repair in Lung Cancer

Asbestos-related lung cancer has been made of a result of various mutations, lesions caused by DNA-damaging ROS [107]. Typically, DNA damage is recognized and repaired by the DNA repair machinery. When DNA repair fails, the genomic integrity of the cell is disrupted and this can lead to cancer. Recent studies have suggested that microRNAs take a one of the important part in the regulation of the DNA repair network (Figure 1) [108,109,110].

An example miR-138 knockdown facilitates DNA damage repair, while miR-138 overexpression inhibits DNA damage repair in small-cell lung cancer (SCLC) cells due to a decrease in the level of H2AX expression, and as a result of miR-138 overexpression, reduction of cell growth and a significant inhibition on cell-cycle progression was detected [111].

hsa-miR-526b also suppressed double-stranded breaks (DSB) repair by inhibition of the Ku80, thus significantly suppressing the NSCLC growth both in vitro and in vivo [112]. Another study demonstrated that inhibition of ATM transcript by miR-27a lead to cell survival and cell cycle progression of A549 cell line [113].

It was shown miR-24-3p plays a role as regulator of the cellular response to DNA damage in pathogenesis of Chronic Obstructive Pulmonary Disease (COPD) which is considered one of the risk factors for lung cancer. Nouws et al. have shown that miR-24-3p suppressed homology-directed DNA repair by inhibition of BRCA1 expression in the cells of parenchymal lung tissue [109].

miR-346 suppressed nucleotide excision repair (NER) by inhibition of the XPC, thus caused promotion tumor growth of A549 cells in xenografts mice [110].

Oncomir miR-34a negatively regulates the process of recombinant repair in NSCLC cells by binding to the 3′-untranslated region of RAD51 [114]. RAD51 expression was also significantly reduced in cells overexpressing hsa-miR-96-5p [115].

Mairinger et all. have shown that miR-125b-5p, miR-21-5p and miR-222-3p expression inversely correlates to PARP1 mRNA expression [108]. Lai et al. showed that miR-7-5p expression levels were significantly reduced in Dox-resistant SCLC cells. miR-7-5p inhibited Dox-induced homologous DSB repair by suppressing the expression of Rad51 and BRCA1 by inhibiting PARP1 [116]. It is interesting, that asbestos activates PARP1 which caused accumulation of single-stranded breaks (SSB) lesions in human mesothelial cells [117]. 

**Table 1:** OncomiRs in lung cancer.

	MicroRNA	MicroRNA Targets and Mechanism	Role in Carcinogenesis	Ref.
1	2	3	4	5
1	cluster of microRNA miR-17-92 (miR-18a, miR-19a, miR-19b, miR-20a and miR-92a)	miR-19a, miR-19b-1 hsa-miR-20a, and miR-92a inhibit translation of the messenger RNA encoding the tumor suppressor PTEN, enhancing cell proliferation and survival. miR-20a targets RNA encoding the E2F2/E2F3 transcription factors, which play a leading role in the regulation of the cell cycle. Repression of the TGF-β antiproliferative signaling pathway: miR-17 and miR-20a target TGF-β-receptor II (TGFBRII), miR-18a targets the participants of this signaling pathway Smad2 and Smad4; miR-18a and miR-19 directly inhibit the antiangiogenic factor thrombospondin-1 (TSP-1).	Proliferation, cell survival, angiogenesis	[35]
2	miR-155	Inhibits translation of the messenger RNA encoding SHIP1 (negative regulator of proliferation) to promote cell growth; C/EBPβ (transcriptional activator for mir-143, which targets one of the main glycolysis enzymes—hk2); TP53INP1 (tumor suppressor regulating autophagy and apoptosis) leads to the inhibition of cell death; MSH2 and MSH6 (key misfit repair proteins) lead to decreased repair; FOXO3 (a transcription factor that regulates genes whose products are involved in apoptosis—for example, Bim and PUMA) leads to avoidance of apoptosis, SOCS1 (negative regulator of cytokine signal transduction) leads to increased proliferation; increases TNF-α levels by binding to the 3′ UTR region of mRNA and increasing transcript stability; with the participation of histone deacetylase HDAC2, represses BRCA1 transcription, which leads to a decrease in repair	Inhibition of apoptosis, proliferation metastases, Warburg effect	[42,43,44]
3	miR-125b	Inhibits translation of the messenger RNAs encoding p53 and proapoptotic proteins Bak1 and Puma; inhibits translation of the messenger RNA encoding the oncosuppressor p14ARF	Avoidance of apoptosis, proliferation	[50]
4	miR-504	Inhibits translation of the messenger RNA encoding p53	Avoidance of apoptosis, proliferation	[49]
5	miR-622	Inhibits translation of the messenger RNA encoding Rb protein	Avoidance of apoptosis	[52]
6	miR -103	Inhibits translation of the messenger RNA encoding proapoptotic protein Bax	Avoidance of apoptosis	[54]
7	miR-197	Inhibits translation of the messenger RNA encoding NOXA and BMF	Avoidance of apoptosis	[55,56]
8	miR-494	Inhibits translation of the messenger RNA encoding PTEN and subsequent activation of the Akt/eNOS pathway	Angiogenic effect	[68]
9	miR-222	Inhibits translation of the messenger RNA encoding cyclin-dependent kinase inhibitor p27Kip1	Proliferation	[46,47]
10	miR-10b	Inhibits translation of the messenger RNA encoding the homeobox D10, which increases expression prometastatic gene RHOC	Lung metastases	[83]
11	miR-652-3p	Inhibits translation of the messenger RNA encoding the Lgl1 protein, which promotes cell adhesion and inhibits cell migration by suppressing the expression of MMP2 and MMP14 and the re-expression of E-cadherin	Metastases	[102]
12	miR-98-5p	Inhibits translation of the messenger RNA encoding TGFBR1	Proliferation, migration and invasion of A549 and H1299 cell lines	[64]
13	miR-27a	Inhibits translation of the messenger RNA encoding ATM	Cell survival and cell cycle progression	[113]
14	miR-346	Inhibits translation of the messenger RNA encoding XPC	Promotion tumor growth	[110]

**Table 2:** Oncosuppressive microRNAs in lung cancer.

	MicroRNA	MicroRNA Targets and Mechanism	Role in Carcinogenesis	Ref.
1	2	3	4	5
1	Let-7	Inhibits translation of the messenger RNAs encoding oncogenes, such as KRAS, NRAS, MYC, HMGA2, and MCT	Inhibits proliferation, inhibits PI3K-mTOR signaling pathway	[37]
2	miR-34	Inhibits translation of the messenger RNA encoding the N-MUS oncogene; cyclin-dependent kinases CDK4 and CDK6; transmembrane receptor protein NOTCH1, involved in the signaling pathway of cancer stem cells; ubiquitin ligase MDMX, involved in p53 degradation; antiapoptotic protein BCL2; sirtuin 1 (SIRT1 gene) involved in p53 degradation; transcription factor E2F3; transcription factors involved in self-renewal of undifferentiated embryonic stem cells: NANOG and SOX2; an integral cellular glycoprotein that plays an important role in cell–cell interactions, cell adhesion, and CD44 migration	Inhibits proliferation, promotes cell cycle arrest and apoptosis	[48]
3	miR-126	Inhibits translation of the messenger RNA encoding S1PR2, thereby inhibiting the PI3K/Akt signaling pathway; inhibits tumor angiogenesis by targeting VEGF-A	Inhibits proliferation and angiogenesis	[38,39]
4	miR-17-5p	Inhibits translation of the messenger RNA encoding TGFßR2, which is significantly increased in NSCLC tissues and cell lines	Inhibits proliferation, causes apoptosis of H460 NSCLC cells	[59]
5	miR-98	Inhibits translation of the messenger RNA encoding PAK1, which promotes NSCLC cell proliferation, migration, and invasion	Inhibits proliferation	[66]
6	miR-128	Inhibits translation of the messenger RNA encoding VEGF-A, vascular endothelial growth factor receptor 2, and VEGFR-3	Inhibits angiogenesis	[69]
7	miR-206	Inhibits the 14-3-3ζ/STAT3/HIF-1α/VEGF pathway	Inhibits angiogenesis	[70]
8	miR-135a	Decreased angiogenesis-related factors VEGF, bFGF, and IL-8	Inhibits angiogenesis	[71]
9	miR-145	Inhibits translation of the messenger RNA encoding mTOR/p70S6K1	Inhibits proliferation	[41,42]
10	miR-646	Inhibits translation of the messenger RNA encoding FGF2 and CCND2	Inhibits proliferation, invasion, and suppress EMT of NSCLC cells in mice	[87]
11	miR-182	Suppresses AKT phosphorylation and accumulation of the Snail transcription factor, which initiates EMT in lung cancer cells. Inhibits translation of the messenger RNA encoding the Met	Promotes the expression of E-cadherin, which leads to inhibition of EMT	[88]
12	miR-7-5p	Inhibits translation of the messenger RNA encoding NOVA2, which disrupts the angiogenesis	Inhibits proliferation, migration, and invasion of tumor	[89]
13	miR-206	Inhibits translation of the messenger RNA encoding the actin-binding protein coronin 1C (CORO1C)	Reduces the ability of cells to grow and metastasize	[91]
14	miR-335-5p	Inhibits translation of the messenger RNA encoding ROCK1	Leads to inhibition of TGF-β1-mediated EMT	[94]
15	miR-126-3p	Inhibits translation of the messenger RNA encoding chemokine receptor 1 (CCR1)	Inhibits NSCLC cell growth and metastasis	[96]
16	miR-192-5p	Inhibits translation of the messenger RNA encoding TRIM44	Reduces migration and invasion	[97]
17	miR-143	Inhibits translation of the messenger RNA encoding Limk1	Suppresses NSCLC cell proliferation, induced apoptosis, and suppresses migration and invasion in vitro	[103]
18	miR-138	Inhibits translation of the messenger RNA encoding H2AX	Inhibition on cell-cycle progression and cell grow	[111]
19	hsa-miR-526b	Inhibits translation of the messenger RNA encoding Ku80	Suppresses NSCLC growth	[112]

## 3. MicroRNAs as Biomarkers of Environmental Factors

As shown in several studies, the microRNA profile can change due to exposure to both chemicals [118,119] and physical environmental factors [120,121,122,123]. Ionizing radiation can cause changes in the microRNA expression profile in human fibroblasts [124] and immortalized cell lines [125]. Changes in the expression of microRNAs under the influence of radiation have a dose-dependent effect [126], which indicates the possibility of using microRNAs as biomarkers of radiation exposure [127]. Cui and colleagues showed that the expression profile of several microRNAs in BEAS2B cells changed upon exposure to radon [128].

We examined 136 subjects, including 49 patients with lung cancer exposed to radon, 37 patients with lung cancer without radon exposure, and 50 volunteers as a control group. The level of free-circulating microRNA hsa-miR-19b-3p was significantly higher in groups of patients with lung cancer compared with healthy individuals. However, no differences were found in the expression level of hsa-miR-19b-3p between patients with radon-induced lung cancer and those who were not exposed to radon. These results indicate that the detection of hsa-miR-19b-3p levels in blood plasma can potentially be used as a noninvasive method for diagnosing lung cancer. However, this microRNA is not suitable as a biomarker for radon exposure [129].

Considering that asbestos is one of the causes of lung cancer and that microRNAs are involved in carcinogenesis, the study of the role of microRNAs in asbestos-induced lung damage is relevant.

## 4. Asbestos and MicroRNA

Extensive research over the past several decades has identified many important pathogenic mechanisms of asbestos fibers for lung cancer; however, the exact molecular mechanisms involved and the cross-linkages between the pathways involved are not fully understood. The significant presence of asbestos in buildings, combined with a long latency period of 30–40 years between exposure and pulmonary toxicity, suggests that asbestos-related lung disease will continue to spread in all countries.

Many studies have examined microRNAs associated with asbestos-induced genetic and epigenetic changes in mesothelioma (highly specific cancer induced by exposure to asbestos) [17,20,130,131,132]. Thus, it was shown that asbestos causes the overexpression of seven microRNAs (miR-374a, miR-24-1, let-7d, Let-7e, miR-199b-5p, miR-331-3p, and miR-96) in lung tumors (Figure 1), and five microRNAs (miR-939, miR-671-5p, miR-605, miR-1224-5p, and miR-202) demonstrate reduced expression under the influence of asbestos [17,21,131,132]. MicroRNAs whose expression profile changes under the influence of asbestos are shown in Table 3.

Santarelli et al. [132] recently established that four microRNAs, namely, miR-126, miR-205, miR-222, and miR-520g, are involved in asbestos-related malignant diseases. Notably, increased expression of miR-126 and miR-222 has been found in asbestos-exposed subjects, and both microRNAs are involved in major pathways associated with cancer development. Epigenetic changes and cross-linking between cancer and stroma can induce miR-126 repression to facilitate tumor formation, angiogenesis, and invasion.

The study included four patient groups, including patients with asbestos and non-asbestos-related non-small cell lung cancer or malignant pleural mesothelioma, as well as healthy subjects. Selected microRNAs were evaluated in the asbestos-exposed population. This study indicated that microRNAs are potentially involved in asbestos-related malignancies, and their expression describes the mechanisms by which microRNAs may be involved in the asbestos-induced pathogenesis of bronchopulmonary diseases [132].

Nymark and coauthors [130] investigated 26 tumors from highly asbestos-exposed and untreated patients and from normal lung tissue control samples that were analyzed for microRNA expression data. They found thirteen microRNAs associated with asbestos, and, among them, eight microRNAs were overexpressed: miR-148b, miR-374a, miR-24-1, Let-7d, Let-7e, miR-199b-5p, miR-331-3p, and miR-96 and five were downregulated: miR-939, miR-671-5p, miR-605, miR-1224-5p, and miR-202. New microRNAs associated with asbestos and histology were identified.

In addition, an inverse correlation of specific target genes was found using an integrative analysis of microRNA and mRNA data from the same patient samples.

A link between the hypermethylation of promotor DNA and inflammation has been shown in many forms of cancer, including asbestos-related lung cancer [133,134]. Molecular genetic analysis have shown changes in the number of DNA copies, changes in the profiles of many microRNAs, and dysregulation of the expression of certain genes in asbestos-associated lung cancer [130,133,134]. However, how asbestos fibers directly or indirectly affect cells in lung cancer and how they interact with cells at the molecular level is currently not known [17].

Asbestos causes pulmonary toxicity, and thus there are several molecular changes: the generation of ROS, which causes tissue and cell damage, apoptosis of alveolar macrophages, and the release of various cytokines and chemokines.

Transcription factors and the tumor suppressor protein p53 are involved in destroying cellular response DNA, causing mitochondrial dysfunction and apoptosis. Activated p53 affects various genes that inhibit cell growth and partially promote apoptosis due to the mitochondrial-regulated pathway of death [135]. The *TP53* oncosuppressor gene, localized on chromosome 17p13, is expressed in all cell types and encodes the p53 protein, which serves as a transcription factor [136]. Several studies have been performed on the genotoxicity and function of *TP53* in the pathogenesis of lung cancer and pleural mesothelioma [136,137,138].

Mitochondrial dysfunction and apoptosis in p53-dependent transcription in an asbestos-induced A549 cell line was determined. When the human papillomavirus E6 protein was transfected to A549 cells, the E6 protein inhibited the function of the p53 gene and lost checkpoint in G1 control. A549-empty vector cells exposed to asbestos revealed dose-dependent mitochondrial dysfunction and apoptosis. Panduri et al. demonstrated that asbestos promoted p53 activity, mRNA levels, protein expression, and translocation of Bax and p53 mitochondria. Inhibitors of p53-dependent transcriptional activation blocked asbestos-induced A549 cell activation of caspase 9 and apoptosis [138].

miR-30d expression levels were decreased in plasma subjects exposed to asbestos, the mesothelial cell line NCI-H2452, and the normal mesothelial cell line MeT-5A treated with chrysotile. After transfection of miR-30d to the NCI-H2452 cell line, a percentage difference in G1/S/G2 stages was not found; however, the overall rate of apoptosis increased [139]. The expression of miR-30d was reduced in NSCLC tissue, and the NSCLC stage I/II was upregulated compared with the NSCLC stage III. Hosseini et al. proposed that this expression level could distinguish between different stages of malignancies [140].

In vitro experiments indicated that miR-30d could attenuate the proliferation and viability of NSCLC cells [141]. The aberrant expression of miR-30a in lung cancer stimulated the expression of myocyte enhancer factor 2D (MEF2D) protein [142]. The myocyte enhancer factor 2 (MEF2) family of human transcription factors, consisting of four subtypes, MEF2-A, -B, -C, and -D, has a diversity of functions in different tissues and has been implicated in numerous diseases. MEF2s play an important role in the activation of the genetic processes that control the cell differentiation, proliferation, and apoptosis in lung cancer. miR-30a prevented the growth and formation of lung cancer cells in a colony by inducing apoptosis [143,144].

Research demonstrated that c-Met, which triggers cell growth in tumor formation, was activated through the suppression of p53-regulated miR-34a in mouse malignant mesothelioma cells [145]. miR-34 also regulated the SNAIL1 gene in EMT in A549 cell lines and targeted an essential p53 tumor suppressor [146]. 

Researchers found that aberrant methylation and silence of miR-34b and miR-34c were observed in asbestos-induced pleural mesothelioma [147]. In lung cancer, the AXL tyrosine kinase receptor is often overexpressed. Via the JNK pathway, the AXL tyrosine kinase receptor activated the ELK1 transcription factor. ELK1, in turn, controlled miR-34a expression by direct activation of the promoter, and miR-34a returned to suppress AXL mRNA. JNK1 overexpression significantly decreased the expression of AXL and caused G1 arrest and apoptosis in lung cancer cells [148]. This allows us to conclude that miR-34 may be involved in the pathogenesis of asbestos-induced lung cancer.

Weber et al. showed that, in the blood of malignant mesothelioma patients exposed to asbestos, miR-20a and miR-103 were downregulated [149]. As we noted above, both of these microRNAs play the role of oncomiRs in the development of lung cancer [35,53], which suggests the presence of different mechanisms of carcinogenesis during the development of asbestos-induced mesothelioma and asbestos-induced lung cancer with the participation of microRNAs. 

It is known that asbestos promotes the generation of ROS, which caused the formation of DSB [150,151]. As a result of DSB, changes occur in the chromatin structure, which leads to phosphorylation of the nucleosomal protein H2A and further damage repair. Msiska et al. showed an increase in the amount of γ-H2AX in human lung cell lines (SAE) and lung adenocarcinoma cells (A549) as a result of asbestos-induced DSB formation [152]. Accumulation of γ-H2AX in both cell lines suggests either impaired DSB repair or long-term production of ROS due to asbestos exposure. It should be noted that the level of γ-H2AX in normal cells was higher than in A549. Histone H2AX plays an important role in apoptosis. Xu et al. showed that knockdown of H2AX in A549 cells affects miR-3196 expression. miR-3196 inhibits apoptosis in A549 cells by acting on the PUMA protein. γH2AX binds to the miR-3196 promoter, inhibits the binding of RNA polymerase II to the miR-3196 promoter, leading to inhibition of miR-3196 transcription [153]. Histone H2AX has been identified as a target for miR-138 [111]. However, there is no information on the change in the expression level of this microRNA due to asbestos exposure. Further research is needed to determine the role of microRNAs involved in DNA repair network in the pathogenesis of asbestos-related lung cancer.

In total, we identified 20 microRNAs, whose expression changed upon exposure to asbestos (Table 3).

**Table 3:** MicroRNAs whose expression profile changed under the influence of asbestos.

	MicroRNA	Expression Level	Type of Cancer	Sample	Ref.
1	miR-374a	Overexpression	Lung cancer	Lung tissue	[130]
2	miR-24-1	Overexpression	Lung cancer	Lung tissue	[130]
3	let-7d	Overexpression	Lung cancer	Lung tissue	[130]
4	let-7e	Overexpression	Lung cancer	Lung tissue	[130]
5	miR-199b-5p	Overexpression	Lung cancer	Lung tissue	[130]
6	miR-331-3p	Overexpression	Lung cancer	Lung tissue	[130]
7	miR-96	Overexpression	Lung cancer	Lung tissue	[130]
8	miR-148b	Overexpression	Lung cancer	Lung tissue	[130]
9	miR-126	Overexpression	Lung cancer	serum	[132]
10	miR-222	Overexpression	Lung cancer	serum	[132]
11	miR-939	down regulation	Lung cancer	Lung tissue	[130]
12	miR-671-5p	down regulation	Lung cancer	Lung tissue	[130]
13	miR-605	down regulation	Lung cancer	Lung tissue	[130]
14	miR-1224-5p	down regulation	Lung cancer	Lung tissue	[130]
15	miR-202	down regulation	Lung cancer	Lung tissue	[130]
16	miR-30d	down regulation	Lung cancer	Lung tissue	[140]
17	miR-34b	down regulation	mesothelioma	serum	[147]
18	miR-34c	down regulation	mesothelioma	serum	[147]
19	miR-20a	down regulation	mesothelioma	blood	[149]
20	miR-103	down regulation	mesothelioma	blood	[149]

**Figure 2:**
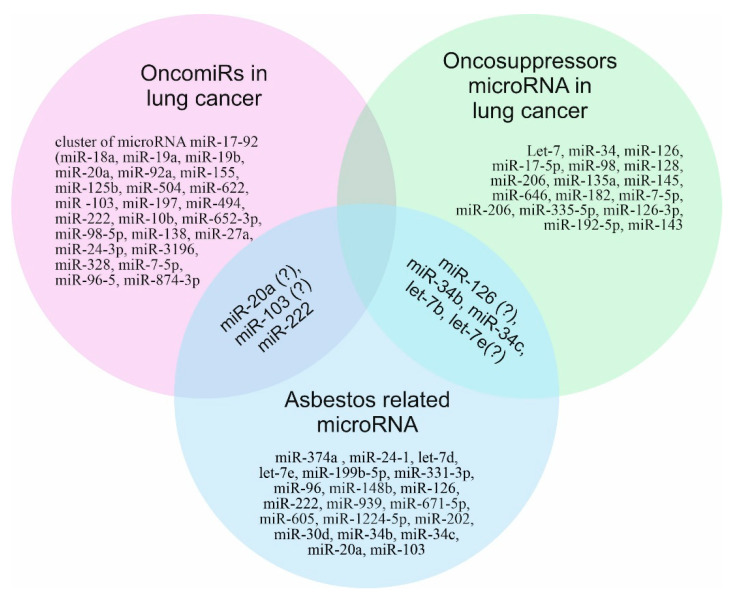
Asbestos-related lung cancer microRNAs.

Comparative analysis of the microRNAs involved in the development of lung cancer and microRNAs whose expression profile changed upon exposure to asbestos made it possible to identify three microRNAs as possible biomarkers of asbestos-induced lung cancer: miR-222, miR-34b, and miR-34c (Figure 2).

Three microRNAs (let-7d, let-7e, and miR-126), which act as tumor suppressors and reduce the risk of developing lung cancer, had an increased level of expression in individuals exposed to asbestos. This indicates the need to study the role of microRNAs in asbestos-induced lung cancer and possibly a different mechanism of carcinogenesis in a malignant different mechanism of carcinogenesis in malignant mesothelioma and lung cancer. 

Most of the reviews, including systematic reviews [154], devoted to the issue of the relationship of asbestos and microRNAs precisely highlight the changes in the microRNA profile in mesothelioma; however, as we can see, this does not always correspond to lung cancer induced by asbestos.

As this review has shown, further studies are needed to answer the question of whether it is possible to use microRNAs as biomarkers of lung damage caused by asbestos for the diagnosis of lung cancer.
